# Analyzing Fluctuation Properties in Protein Elastic Networks with Sequence-Specific and Distance-Dependent Interactions

**DOI:** 10.3390/biom9100549

**Published:** 2019-09-30

**Authors:** Romain Amyot, Yuichi Togashi, Holger Flechsig

**Affiliations:** 1 Department of Mathematical and Life Sciences, Graduate School of Science, Hiroshima University, 1-3-1 Kagamiyama, Higashi-Hiroshima, Hiroshima 739-8526, Japan; romain-amyot@hiroshima-u.ac.jp; 2 Research Center for the Mathematics on Chromatin Live Dynamics (RcMcD), Graduate School of Integrated Sciences for Life, Hiroshima University, 1-3-1 Kagamiyama, Higashi-Hiroshima, Hiroshima 739-8526, Japan; 3 RIKEN Center for Biosystems Dynamics Research (BDR), 3-10-23 Kagamiyama, Higashi-Hiroshima, Hiroshima 739-0046, Japan; 4 Cybermedia Center, Osaka University, 5-1 Mihogaoka, Ibaraki, Osaka 567-0047, Japan; 5 Nano Life Science Institute (WPI-NanoLSI), Kanazawa University, Kakuma-machi, Kanazawa, Ishikawa 920-1192, Japan

**Keywords:** protein fluctuations, coarse-grained modeling, elastic networks, B-factors, sequence specificity

## Abstract

Simple protein elastic networks which neglect amino-acid information often yield reasonable predictions of conformational dynamics and are broadly used. Recently, model variants which incorporate sequence-specific and distance-dependent interactions of residue pairs have been constructed and demonstrated to improve agreement with experimental data. We have applied the new variants in a systematic study of protein fluctuation properties and compared their predictions with those of conventional anisotropic network models. We find that the quality of predictions is frequently linked to poor estimations in highly flexible protein regions. An analysis of a large set of protein structures shows that fluctuations of very weakly connected network residues are intrinsically prone to be significantly overestimated by all models. This problem persists in the new models and is not resolved by taking into account sequence information. The effect becomes even enhanced in the model variant which takes into account very soft long-ranged residue interactions. Beyond these shortcomings, we find that model predictions are largely insensitive to the integration of chemical information, at least regarding the fluctuation properties of individual residues. One can furthermore conclude that the inherent drawbacks may present a serious hindrance when improvement of elastic network models are attempted.

## 1. Introduction

Proteins are involved in most cellular processes. Their functions are often accompanied by conformational motions which can have timescales ranging from picoseconds (atomic group fluctuations), nanoseconds (collective movement of residue groups), to micro- and even milliseconds (relative domain motions). Slow dynamics cannot typically be followed in atomistic molecular dynamics (MD) simulations, despite the use of supercomputers. Instead, coarse-grained models are often employed [1,2]. In particular, elastic network models (ENMs) are simple and hence widely used [3,4,5,6].

In a protein elastic network, beads represent amino-acid residues and are connected by elastic springs, effectively taking into account potential interactions between them. The success of ENMs is related to their ability to reproduce well the pattern of residue displacement in protein structures due to thermal fluctuations (B-factors). In the original formulation, and in most current applications too, a uniform interaction strength is considered for all residue pairs, i.e., all elastic springs have the same stiffness which is used to scale B-factors. In a plethora of studies, this assumption was lifted aiming to improve ENM predictions, e.g., by iteratively changing stiffness constants to optimize correlation between experiment and model [7], or by introducing a dependence on the distance between residues in the reference structure [8,9,10,11]. Elastic network potentials have been also validated and improved using atomistic MD simulations [12,13,14].

Recently, based on the analysis of a large set of protein NMR structures, elastic network force constants which are specific to the amino acids of residue pairs, and also include a dependence on the distance, have been extracted [15]. This approach appears to be appealing, since first, sequence-dependent interactions have a plausible physical interpretation, and, second, involving very soft far-ranged interactions seems to naturally resolve the common problem of a fixed cutoff distance for residue contacts in protein elastic networks.

While in the original publication [15] an improvement of the new model variants was demonstrated as a result of averaging over a large set of protein structures, the results obtained for individual proteins were not presented. Therefore, the interpretation of how the quality in model predictions becomes enhanced and what the underlying origins are remains incomplete.

We aimed to fill this gap by applying the novel model variants to a specific set of protein structures which have been considered also in several previous publications of protein ENMs [16,17,18]. The emphasis was on how well residue fluctuations obtained in experiments were reproduced by the different models. We then extended our investigation to a very large set of protein structures to systematically explore the performance of the new models and compare them to traditional anisotropic network models (ANMs). We eventually discuss intrinsic drawbacks which present a serious hindrance when improvements of elastic network models, such as integrating chemical information, are to be evaluated.

## 2. Elastic Network Normal Mode Analysis

For our analysis, we employ the anisotropic network model of proteins [19,20]. The elastic energy of the network with *N* beads is

(1) $$\begin{array}{c} U=\frac{1}{2}\sum_{i<j}^{N}\kappa_{ij}\left(s_{ij},d_{ij}^{\left(0\right)}\right)\cdot {\left(d_{ij}-d_{ij}^{\left(0\right)}\right)}^{2}, \end{array}$$

where $d_{ij}=\left|R_{i}-R_{j}\right|$ is the length of the spring connecting beads *i* and *j* at positions $R_{i}$, $R_{j}$, and $d_{ij}^{\left(0\right)}=\left|R_{i}^{\left(0\right)}-R_{j}^{\left(0\right)}\right|$ is the natural spring length. Equilibrium positions $R_{i}^{\left(0\right)}$ coincide with C${}_{\alpha }$-atom positions of residues. The stiffness $\kappa_{ij}$ of a spring can depend on the combination of amino-acid residues $s_{ij}$ of the bead pair (i,j) and the natural length $d_{ij}^{\left(0\right)}$.

Note that the energy has a complex and non-linear dependence on the spatial coordinates of bead positions. However, near the equilibrium state of the network, the contributions which are quadratic in the displacements of bead positions are dominant, and network equations of motion reduce to $A^{T}HA=\Lambda$. Here, H is the 3N×3N Hessian matrix with elements $H_{i,\alpha ;j,\beta }=\partial^{2}U\left(R_{1},\cdots ,R_{N}\right)/\left(\partial R_{i,\alpha }\partial R_{j,\beta }\right){|}_{R=R^{\left(0\right)}}$ (for α,β=x,y,z), $A={\left[a^{\left(1\right)},\cdots ,a^{\left(3N\right)}\right]}_{3N\times 3N}$, and $\Lambda =\mathrm{diag}(m\omega_{1}^{2},\cdots ,m\omega_{3N}^{2})$. Near the equilibrium, network dynamics corresponds to 3N independent vibrations (modes) with bead displacements $a^{\left(k\right)}$ in mode *k* (the eigenvectors of H) having frequencies $\omega_{k}$ (roots of H’s mass-scaled eigenvalues). Six of these modes are trivial and correspond to global translations and rotations. Fluctuations in the positions of network beads are described by a superposition of all modes. The mean-square fluctuations are given by MSF${}_{i}=\langle |R_{i}\left(t\right)-R_{i}^{\left(0\right)}{|}^{2}\rangle =\left(k_{B}T/m\right)\sum_{k=7}^{3N}{\left|a_{i}^{\left(k\right)}\right|}^{2}/\omega_{k}^{2}$ for bead *i*. The corresponding B-factor predicted by the model is obtained as $B_{i}^{\mathrm{pred}}=\left(8\pi^{2}/3\right)\cdot$MSF${}_{i}$.

In our study, we compare B-factor predictions from different model variants. For the conventional anisotropic network model with a distance cutoff $l_{c}$ (termed ANM${}_{l_{c}}$), the spring stiffness in Equation (1) is $\kappa_{ij}=\kappa \cdot \Theta \left(l_{c}-d_{ij}^{\left(0\right)}\right)$, and only those residues which are separated by a distance less than $l_{c}$ in the equilibrium network conformation interact (Θ is the Heaviside step function). For the sequence-specific model with a cutoff distance (sANM${}_{l_{c}}$), we have $\kappa_{ij}=\kappa \left(s_{ij}\right)\cdot \Theta \left(l_{c}-d_{ij}^{\left(0\right)}\right)$, and combined with a distance dependence (sdANM), $\kappa_{ij}=\kappa \left(s_{ij},d_{ij}^{\left(0\right)}\right)$, respectively. In the two latter cases, we extract the stiffness constants from tables provided in [15] (see also Appendix A.1).

### 2.1. Comparison to Experiments

To score the accuracy of model predictions, the Pearson correlation coefficient between predicted and experimental B-factors was computed

(2) $$\begin{array}{c} PCC=\frac{\sum_{i=1}^{N}\left(B_{i}^{\mathrm{pred}}-{\bar{B}}^{\mathrm{pred}}\right)\left(B_{i}^{\exp}-{\bar{B}}^{\exp}\right)}{\sqrt{\sum_{i=1}^{N}{\left(B_{i}^{\mathrm{pred}}-{\bar{B}}^{\mathrm{pred}}\right)}^{2}}\sqrt{\sum_{i=1}^{N}{\left(B_{i}^{\exp}-{\bar{B}}^{\exp}\right)}^{2}}}. \end{array}$$

Since elastic network stiffness constants are defined only up to a constant scaling factor, we can rescale for each protein the predicted B-factors such that ${\bar{B}}^{\mathrm{pred}}={\bar{B}}^{\exp}$, i.e., the corresponding average values become equal. This was important when predicted and experimental B-factor patterns were directly compared, either visualizing them or by determining the deviation for each residue during the systematic analysis of the large protein data set.

### 2.2. Set of PDB Structures

For the systematic evaluation of predictions from the different elastic networks, we have chosen a large set of PDB entries which obeyed the following conditions. Only X-ray crystallographic structures characterized as proteins with no nucleic acids bound and which had a resolution of 2 Å or better were considered. All structures which had missing residues or unconventional amino-acid types in their sequence were further omitted. After this pre-selection, the elastic network for each PDB file was constructed for the structure corresponding to the crystallographic asymmetric unit. If further modes with vanishing eigenvalues, in addition to the trivial 6 zero-modes, were found in the energy spectrum of the elastic network (indicating the presence of less-sufficient or disconnected network regions), the corresponding PDB file was rejected. The numbers of eventually accepted structures were 2009 for ANM${}_{10}$ and sANM${}_{10}$, 2038 for ANM${}_{13}$ and sANM${}_{13}$, and 2040 for ANM${}_{16}$ and sdANM. See Appendix A.2 for further details.

### 2.3. Data Analysis

We have analyzed how residue fluctuations within a protein elastic network are related to the network connectivity. Since the network architecture is determined by the cutoff distance for residue interactions, which is different for the considered models, we had to specify a relative connectivity for each residue bead within a given protein elastic network. It was defined as the ratio of the degree of each bead, i.e., $D_{i}=\sum_{j\neq i}^{N}\Theta \left(l_{c}-d_{ij}^{\left(0\right)}\right)$, and the maximum degree found in the same network, i.e., $\max\{D_{i}\}$.

The criteria which were applied to classify the chemical specificity of a residue bead and its location within secondary structural motifs are provided in the Appendix A.5.

## 3. Results

We have compared traditional anisotropic network models (ANM) and recently proposed model variants [15] which include interactions that are sequence-specific and can additionally depend on the distance (sANM and sdANM). Each model was applied to a specific set of proteins, and its performance was evaluated by how well fluctuation dynamics reproduced the experimentally known pattern of B-factors (see previous section). We could then compare results obtained for a specific protein but also extract information about the origin of differences in the predictions of model variants.

In particular, we have compared the sequence-specific models sANM${}_{10}$ and sANM${}_{13}$, which impose a cutoff distance of 10 Å and 13 Å for interactions between network beads, to their traditional counterparts ANM${}_{10}$ and ANM${}_{13}$. The heterogeneity of sequence related stiffness constants in those models ranges from values between 0.226 for glycine-glycine pairs to 2.348 for isoleucine-valine pairs, which corresponds to a dispersion of one order of magnitude as compared to the homogeneous stiffness constants in ANMs. The stiffness constant between neighboring residues in the protein backbone is artificially set to 10. The model variant sdANM includes both a dependence on sequence and the equilibrium distance between beads, and stiffness constants range from $10^{-3}$ for very soft far-ranged interactions to very stiff contacts between neighboring residues in the protein backbone, with the constant 43.52. Since in this model all pairs of residue beads which are separated by an equilibrium distance larger than 16.5 Å have zero interaction strength, we decided to study also the ANM with 16 Å cutoff distance (ANM${}_{16}$). However, when comparing sdANM to the ANM models, it should be noted that the heterogeneity of stiffness constants stems from a combination of sequence and distance dependence, while the distinction of ANMs is solely related to the different cutoff distance and all interactions have homogeneous strength.

**Figure 1:**
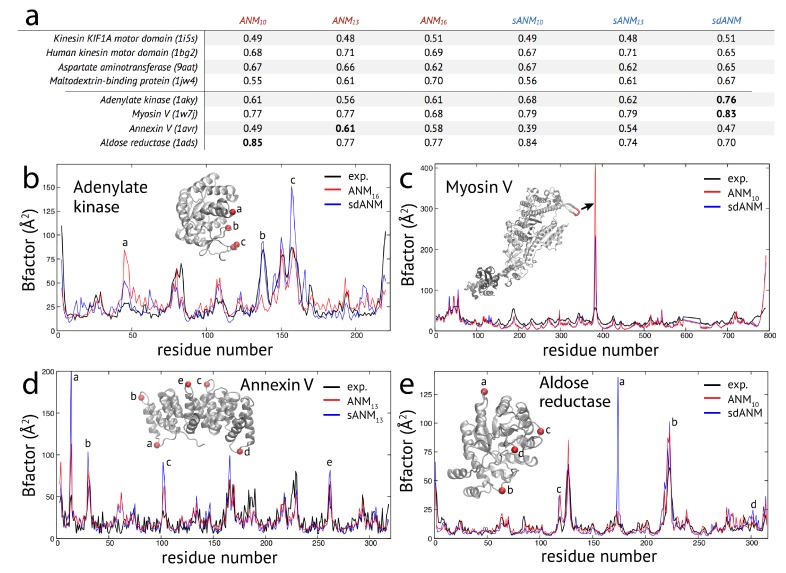
Fluctuation dynamics in studied proteins. (**a**) Table of correlation coefficients for protein structures obtained from the considered model variants. For each of the two identified groups, four examples are shown. In the second group, bold numbers indicate for each protein the highest correlation of experiment and model predictions. (**b**–**e**) B-factor profiles for four protein structures are shown. Model predictions are displayed as red and blue lines; corresponding experimental data is shown in black. In the B-factor profiles, positions where model estimates are significantly poor were marked for each protein. The corresponding regions in the respective protein structure are also indicated.

The results obtained for the studied set of protein structures can be separated into two groups. In one group of proteins, the correlation coefficient for agreement between model and experiment was very similar when comparing sequence-dependent variants to ANMs. Representative examples from this group are shown in the table in Figure 1a. Changes in the correlation were not larger than 0.06 between the employed models. An exception is the maltodextrin-binding protein, where an improvement from 0.55 for ANM${}_{10}$ to 0.61 for ANM${}_{13}$ and further to 0.70 for ANM${}_{16}$ was seen. For the sequence-specific variants, we found 0.56 for sANM${}_{10}$, 0.62 for sANM${}_{13}$ and 0.67 for sdANM. Hence, this improvement is related to a change in the cutoff distance for interactions, i.e., in the network architecture, but not to the presence of sequence information.

In the second group of protein structures, we found notable differences between predictions by the new model variants and traditional ANMs. The results for a selected set of structures are shown in the table in Figure 1a. For those proteins, the highest correlation coefficient for agreement between model and experiment was at least 0.7, or the difference between poorest and best correlation was larger than 20%.

In the case of adenylate kinase, both sANM models yield higher correlations than the ANMs and the highest value 0.76 was obtained with sdANM, corresponding to an improvement of 0.15 compared to the best performing ANM. The profile of B-factors (see Figure 1b) shows that large deviations between model predictions and experiment are found at positions which correspond to irregular secondary structures in this protein, mostly loops in the lid domain. sdANM improves predictions in some of those parts, but performs worse than ANM in other parts. In the myosin V motor protein, we find overall high correlation values (0.68 to 0.79) and negligible differences between sANM and ANM, indicating no sensitivity with respect to sequence specificity. However, sdANM showed a further increase in correlation to 0.83. As can be clearly seen in Figure 1c, this improvement can be assigned to a few residues of the highly flexible HCM loop. In ANMs, their fluctuations are massively overestimated, likewise still in sdANM, but less. In the case of the annexin V protein, the best correlation of 0.61 is found for ANM${}_{13}$. The same model with sequence information sANM${}_{13}$ showed a decrease to 0.54, which originated from a systematic over-estimation of B-factors in loop regions connecting adjacent alpha-helices (see Figure 1d). In the aldose reductase model, predictions were in well agreement with experiment (correlations 0.70 to 0.85 for ANM${}_{10}$) and there was marginal difference between ANM and the corresponding sANM model. Interestingly, for sdANM, the correlation dropped to 0.7 which in this protein results from a massive over-estimation of fluctuations of a single loop, which are very well reproduced by ANM${}_{10}$. Besides this local effect, the corresponding B-factor profiles are otherwise very similar (see Figure 1e).

**Figure A1:**
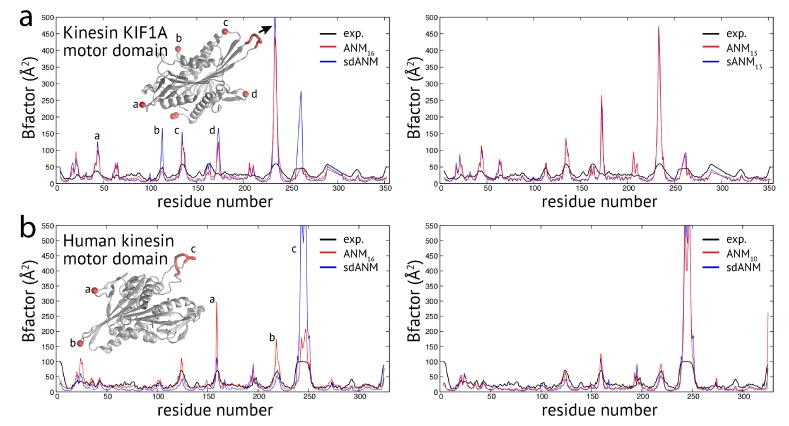
Fluctuation dynamics in studied proteins. B-factor profiles for two kinesin protein structures are shown; the motor domain of (**a**) kinesin KIF1A and of (**b**) human kinesin. Model predictions are displayed as red and blue lines; corresponding experimental data is shown in black. In the B-factor profiles, positions where model estimates are significantly poor were marked for each protein. The corresponding regions in the respective protein structure are also indicated.

Proceeding with the observations made for proteins in the second group, we wanted to check whether effects related to flexible regions were already present for examples from the first group, where the variation in predictions by the different models was minor (except for the discussed cutoff-related cases). In the case of the kinesin KIF1A motor domain, the correlation coefficients between model predictions and experiments were the same for sdANM and ANM${}_{16}$ (0.51). The corresponding pattern of B-factors (shown in Figure A1a) shows that large deviations between experiment and the models can be directly associated with the multiple loop regions inside this domain. There the models massively over-predict fluctuations, which explains the overall poor agreement with the experiment. It is interesting to note that the pattern obtained with the sequence-specific model sANM${}_{13}$ is almost identical to that corresponding to the traditional model ANM${}_{13}$ without sequence information (see Figure A1a). For the human kinesin motor domain, we also found the overall well agreement of model and experiment (e.g., 0.68 for ANM${}_{10}$, 0.69 for ANM${}_{16}$ and 0.65 for sdANM) to be nonetheless significantly suffering from over-predictions in the fluctuations of loop regions (see Figure A1b). We noted in this example that the distance-sequence-specific model sdANM and the traditional model with a small cutoff distance ANM${}_{10}$ have a similar B-factor pattern (see Figure A1b).

**Figure 2:**
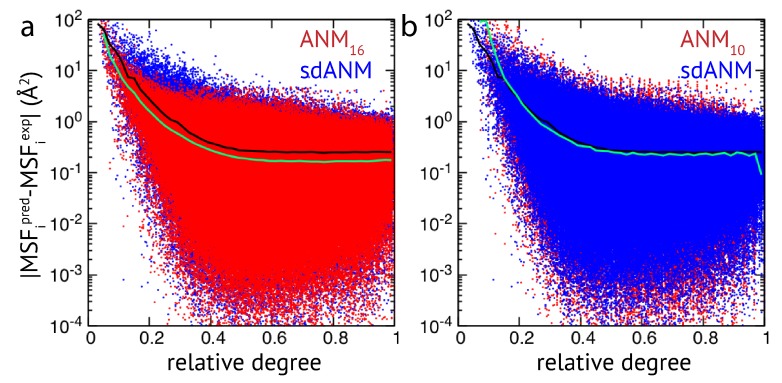
Extensive analysis of predictions by ENM variants. The error in MSF prediction for a set of more than 2000 protein structures is shown (in logarithmic scale). Each data point represents the absolute deviation of predicted and experimental MSF obtained for a single residue bead in a protein elastic network, as a function of its relative degree. (**a**) Red dots are obtained for ANM${}_{16}$ and blue dots correspond to sdANM predictions. (**b**) ANM${}_{10}$ is compared with sdANM. In both plots, the average absolute errors are indicated as black lines for sdANM and as green lines for ANMs.

The results obtained by us for a relatively small set of proteins indicate that the quality of how well residue fluctuations are predicted by elastic network models appears to be intrinsically linked to poor estimations in highly flexible protein regions. While we originally attempted to better understand how sequence-specific elastic networks can improve model predictions, we found this drawback to be present in the traditional ANMs as well as in the considered proposed model variants with sequence specificity and distance dependence. Those results point towards a generic problem of elastic network models which we will refer to in the Discussion section. In the context of how newly proposed models compare to traditional models, we wanted to go beyond a small set of proteins and further explore the observed effects in a systematic way by applying all different models to a large set of proteins (>2000 structures) to compare their predictions with corresponding experimental data. The focus of our analysis was to relate the agreement between model and experiment to the connectivity within an elastic network. For details on the chosen set of PDB entries and the analysis of data, we refer to the Methods section. Our results show that fluctuations of weakly connected network residues are intrinsically prone to be wrongly predicted by all models, and poorly connected parts are most vulnerable to over-estimation. In Figure 2, results of sdANM are shown in comparison with those from ANMs. For each residue bead within a protein elastic network, the deviation of the MSF predicted by the model and the corresponding experimental value (i.e., the prediction error) is displayed as a function of its relative connectivity (see Methods), taking into account more than 2000 different structures.

**Figure A2:**
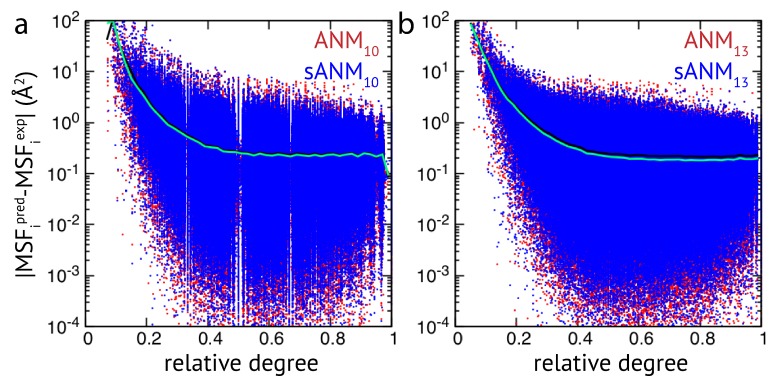
Extensive analysis of predictions by ENM variants. The error in MSF prediction for a set of more than 2000 protein structures is shown (in logarithmic scale). Each data point represents the absolute deviation of predicted and experimental MSF obtained for a single residue bead in a protein elastic network, as a function of its relative degree. (**a**) Red dots are obtained for ANM${}_{10}$ and blue dots correspond to sANM${}_{10}$ predictions. (**b**) ANM${}_{13}$ is compared with sANM${}_{13}$. In both plots, the average absolute errors are indicated as black lines for sANMs and as green lines for ANMs.

**Figure A3:**
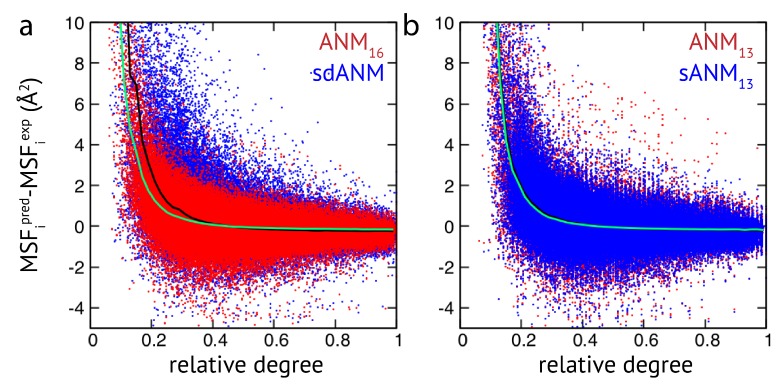
Extensive analysis of predictions by ENM variants. The error in MSF prediction for a set of more than 2000 protein structures is shown. Each data point represents the deviation of predicted and experimental MSF obtained for a single residue bead in a protein elastic network, as a function of its relative degree. (**a**) Red dots are obtained for ANM${}_{16}$ and blue dots correspond to sdANM predictions. (**b**) ANM${}_{13}$ is compared with sANM${}_{13}$. In both plots, the average errors are indicated as black lines for sANMs and as green lines for ANMs.

Even in elastic networks with a large cutoff distance (ANM${}_{16}$), fluctuations of network beads with a small relative connectivity (<0.2) are predominantly over-predicted to a significant extent, and those of poorly connected beads are massively over-predicted (see Figure 2a and Figure A3). As we show, the model with sequence- and distance-specific interaction (sdANM) performs on average worse than ANM${}_{16}$ over the entire range of network connectivities. The error became increased in the region of very poor bead connectivity. The reason may be that the long-ranged interactions which are very soft in sdANM effectively increase the occurrence of such poorly connected beads, and the effect of mispredictions thus becomes enhanced (see Discussion section). Interestingly, the performance of sdANM on the average closely resembles that of elastic networks with a relatively small cutoff distance of 10 Å (see Figure 2b). When comparing the sequence-specific models sANM${}_{10}$ and sANM${}_{13}$ to their corresponding traditional ANM counterparts ANM${}_{10}$ and ANM${}_{13}$, we could not obtain any notable differences in their performance (see Figure A2 and Figure A3). Moreover, as above mentioned, in all models fluctuations of those residues which correspond to weakly connected network regions are prone to be massively over-predicted.

In the last part of our study, we have used the systematic analysis of protein elastic networks to investigate how model predictions may depend on the chemical nature of the residues and on their location within certain secondary structures or unstructured regions within the protein. The motivation for this was that interactions in sequence-specific models implicitly include chemical information of the involved amino-acid residues. Moreover, as revealed by our results for the smaller set of individual proteins, mis-prediction of fluctuations appeared to be particularly severe in loop-like regions. The same set of protein elastic networks used for the above analysis was reanalyzed and the prediction error for each residue bead was computed.

**Figure 3:**
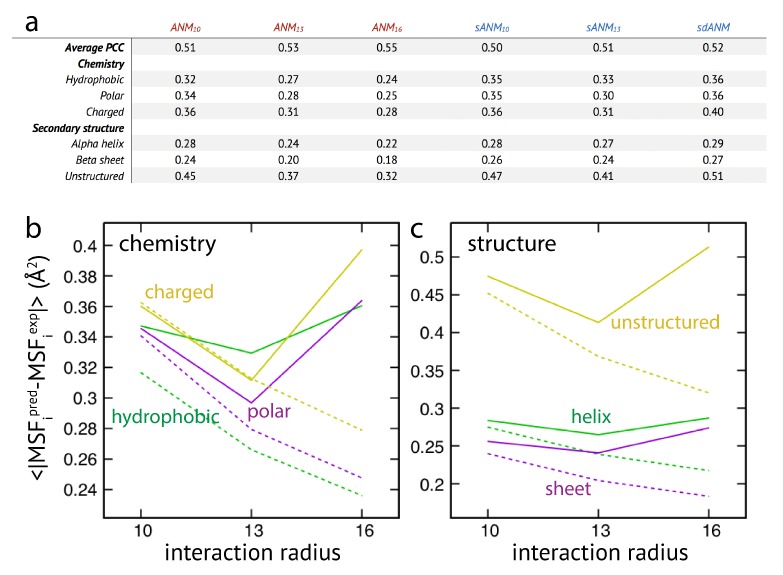
Extensive analysis of predictions by ENM variants. (**a**) The correlation coefficient averaged over all considered structures is shown for all models (top row). The averaged absolute deviation in MSF predictions for residue beads from groups with different chemical specificity (hydrophobic, polar, and charged) is shown for all models. Also shown is the averaged absolute error for residue beads categorized into different secondary structural elements. In (**b**,**c**), corresponding graphs are shown. Solid lines correspond to sANM data, where values for sdANM are displayed at interaction radius abscissa position 16 Å. Dashed lines are used for ANM data.

The results are shown in Figure 3. We first note that on average the agreement between model predictions and experiment is of mediocre quality. Correlation coefficients for all models range between 0.51 to 0.55 and are lower as compared to values given in the original publication [15]. We find that on average the difference between sequence-specific and non-specific traditional models is marginal, in agreement with [15].

Regarding the chemical specificity (see Figure 3), averaged prediction errors by sequence-specific models sANM${}_{10}$, sANM${}_{13}$ and their corresponding counterparts ANM${}_{10}$, ANM${}_{13}$ are similar for polar and charged residues. There is, however, a difference for hydrophobic residues, where the prediction error by sequence-specific models is on average larger when compared to ANMs (by 10% for sANM${}_{10}$ and by 24% for sANM${}_{13}$). We will refer to this aspect in the Discussion section. For the sdANM model, average errors for all residue types were larger than those obtained with all other models, and largest for charged residues. The classical model with a large cutoff ANM${}_{16}$ performed best among all models with the average errors reduced by more than 20% for all residue types as compared to other ANMs.

When model predictions are related to the secondary structural motif within a protein, we found that, on average, predictions for residue beads belonging to alpha-helices and beta-sheets were systematically better as compared to those located in unstructured regions (see Figure 3). In the latter case, the average prediction error was much larger for all considered models. This agrees with our previous observations, and strengthens the conclusion on the intrinsic vulnerability of flexible regions in predictions by ENMs (see Discussion section). It is furthermore notable that regardless of the structural motifs, average prediction errors by the sequence-specific models are throughout larger when compared to the classical ANMs. It is also not to our surprise that the classical model with a large cutoff of 16 Å produced the least prediction errors on average (reduced by more than 20% as compared to other ANMs). The sdANM model, in contrast, performed worst among all models (see Figure 3).

Graphs in Figure 3b,c display the cutoff-related improvement of predictions in ANMs (and sANMs). In ANMs, this effect is particularly pronounced for unstructured protein regions (and for all chemical types), while it is much less seen for helix and sheet regions. This shows that improvements result from rigidification within networks, decreasing the frequency of highly flexible parts. In sdANM, which imposes a large cutoff of 16.5 Å, predictions of unstructured regions (and for all chemical types) are on average much worse compared to ANM${}_{16}$. In sdANM, rigidification of network structures is absent due to the softness of long-range interactions, and the frequency of poorly connected problematic parts is enhanced.

For the extensive analysis of a large data set of proteins, we preferred X-ray crystallographic structures, for their greater presence in the Protein Data Bank as well as the availability of experimental B-factors. However, the new sequence- and distance-dependent models were constructed based on the analysis of NMR data. In fact, in the original publication, the improved performance of the sdANM model was demonstrated for a set of NMR protein structures [15]. While we found the performance of sdANM to be the poorest in reproducing experimental B-factors, we wanted to complement our study by considering also the application of different model variants to a smaller set of protein structures which are available from solution NMR experiments (obviously without aiming to repeat the previous study). It is important to note that the interpretation of NMR experimental data is much different from that of crystallographic data; instead of B-factors, conformational variation among the NMR models is assumed as the index of structural fluctuations here, in the same way as in preceding studies [15,21,22]. Therefore, a direct comparison of model performances for the different data sets must be treated with caution. We explain the choice of the protein data set and the methods to compare model predictions with NMR experimental data in the Appendix A.3 and Appendix A.4.

**Figure A4:**
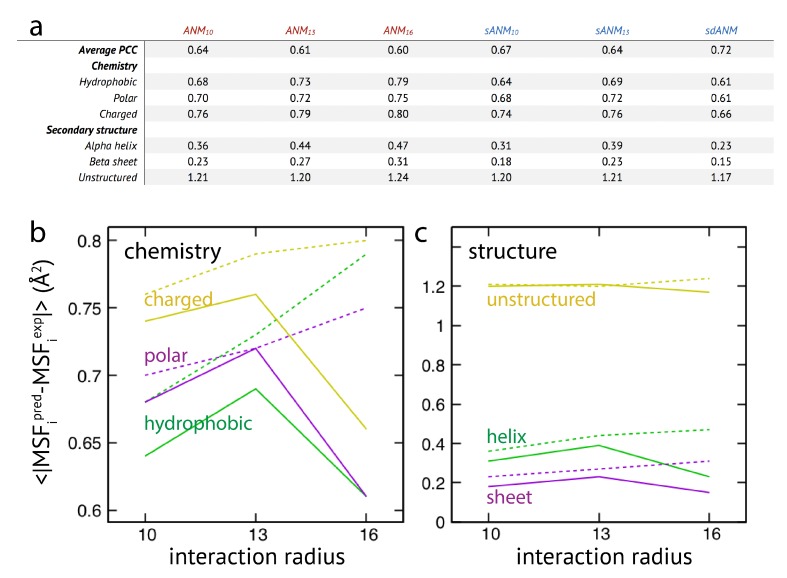
Analysis of predictions by ENM variants for the NMR structural data set. (**a**) The correlation coefficient averaged over all considered proteins is shown for all models (top row). The averaged absolute deviation in MSF predictions for residue beads from groups with different chemical specificity (hydrophobic, polar, and charged) is shown for all models. Also shown is the averaged absolute error for residue beads categorized into different secondary structural elements. In (**b**,**c**), corresponding graphs are shown. Solid lines correspond to sANM data, where values for sdANM are displayed at interaction radius abscissa position 16 Å. Dashed lines are used for ANM data.

The results of our analysis are shown in Figure A4. We find that, based on the average correlation coefficient between the model predictions and experiments, the inclusion of sequence information into the cutoff-based elastic network has a marginal effect, whereas the model with additional distance-dependent interactions (sdANM) shows the highest correlation on average. This agrees with the observation from the original publication [15]. Our results show that the superior performance of sdANM primarily stems from a better prediction of the motion of stable secondary structural motifs (alpha-helices and beta-sheets). The quality of the same model, however, suffers from the mis-prediction of the dynamics of very flexible unstructured regions. This drawback is found among all considered models, the traditional ANMs as well as the sequence- and distance-dependent new models, and its presence seriously hampers the quality of elastic networks to correctly resolve conformational dynamics seen in experiments; which is similar to the conclusion drawn from the analysis of crystallographic protein structure data.

## 4. Discussion

We have studied the properties of residue fluctuations in recently proposed sequence-specific and distant-dependent protein elastic networks, and compared their predictions with those of conventional anisotropic network models. We first aimed to provide an interpretation of how new descriptions may enhance model predictions, by applying them to a specific set of classically studied proteins. We then found that predictions by the new models became improved for some proteins, while the agreement with experiments dropped in other cases.

This ambiguity originates from ubiquitously present false predictions of residue fluctuations in highly flexible protein regions. In the elastic network model, those regions correspond to poorly connected parts, which are highly vulnerable to incorrect predictions. Through a systematic analysis of more than 2000 protein structures, we have shown that this drawback is present in all the considered model variants and intrinsically affects predictions.

Our analysis furthermore reveals several systematic drawbacks of the new models which are worth mentioning. (i) False prediction of motions in flexible protein regions is enhanced in the sequence and distance-specific sdANM model. The reason is that the softening of long-ranged interactions, reinforced by the stiffening of short-ranged interactions in this model, effectively increases the occurrence of poorly connected residue beads. (ii) On the average, the performance of sdANM resembles that of a traditional ANM with a small cutoff distance. (iii) Related to the chemical nature of amino acids, fluctuation predictions of hydrophobic residues by the sequence-specific models are prone to disagree with X-ray experimental data of protein structures. The sequence-dependent stiffness constants have been determined for elastic networks corresponding to solution NMR conformational ensembles, where the presence of hydrophobic core residues is taken into account. In crystal structures, however, this aspect is often not correctly reproduced, e.g., in a situation when only parts of a larger protein complex became determined in isolation.

Inherent shortcomings in the prediction of residue fluctuations by anisotropic elastic network models have been previously discussed. In particular, problems related to structural components which protrude out of a globular protein structure (e.g., loops) have been identified and referred to as the “tip effect” [23]. It describes motions with abnormally large magnitudes which can occur near those regions at comparably little cost, due to sparser network connectivity. It has been shown that a model extension which, in addition to changes in spring lengths, also penalizes angular deviations of the protein backbone can dampen the “tip effect” through stiffening of too soft regions and improve predictions [23,24,25].

A separate study has also shown that mean-square fluctuations of residues at the surface of a protein are systematically overestimated [26]. False predictions related to hydrophobic residues and those located in less structured protein regions (loops and turns) have also been observed. It was furthermore concluded that fluctuation properties are rather insensitive to the chemical properties of amino-acid residues. A similar observation was made when chemical information of residues was included into the interactions of Gaussian elastic network models (GNM) of proteins [27]. Robustness of elastic network performance with respect to variations in the spring constants has been also demonstrated based on comparison with atomistic MD simulations [14].

On the other side, generic limitations in the accuracy of B-factor patterns determined from X-ray crystallography are a well discussed issue. In particular, perturbations generated by the crystal packing should affect the mobility of more flexible residues, located e.g., at the surface of a protein. Including molecular contacts in the crystal into the model has been demonstrated to improve agreement of GNM predictions with experiments [10,17]. Similar attempts have been made also for ANM [10,28,29] (see also [30]). The effect of crystal packing on internal protein motions has been taken into account also in an extension of GNM which introduced variable weights for the amplitudes of normal modes [31].

Although not as systematic as the case of crystallographic structures above, to assess the performance of these models in reproducing structural fluctuations observed in solution NMR, we additionally analyzed 132 protein NMR structures. We indeed found that sdANM reproduces fluctuations in stable secondary structures such as alpha-helices and beta-sheets better than ANMs, supposed to be benefitted more from the distance-dependence rather than the sequence-dependence of the model’s parameters. The problem related to predictions of very flexible regions however persisted, as shown by the large prediction errors for the unstructured regions. In ENMs, the solvent environment is usually not taken into account, and hence, possible damping effects are ignored. Since the new sequence- and distance-dependent ENM stiffness constants have been extracted from the analysis of solution NMR conformational ensembles, they should already include some effective contribution by the solvent. Nonetheless, our results suggest that, to counter the “tip effect”, additional energy barriers should be introduced to restrict otherwise too soft motions of protruding protein parts.

While previous works mentioned the liability of elastic networks to correctly reproduce fluctuations of very flexible protein regions, our study provides a systematic exploration of such inherent drawbacks. We have also demonstrated that recently proposed models which include sequence- and distance-dependent interactions do not resolve such problems; they may even be enhanced. Beyond these effects, our results are another demonstration that predictions of elastic network models are rather insensitive to the integration of chemical information, strengthening the picture that fluctuation properties of a protein are largely determined by the architecture of its corresponding elastic network (representing its stable folded conformation).

A further conclusion from our study is that the inherent drawbacks generally present a serious hindrance when improvement of elastic network models is attempted. In this study, the comparison of models was based on the evaluation of fluctuation magnitudes of individual residues. A more complete picture can in principle be obtained by considering the direction of fluctuations and their correlations between different sites (see e.g., [22,32,33]). However, those measures would be affected by the intrinsic shortcomings too. Indeed, we have also attempted to compare the prediction of directional residue fluctuations between traditional ANMs and the new sequence- and distance-dependent models, but found the results to be contaminated by the “tip effect” too. Though a comparison with anisotropic B-factors obtained from very high-resolution experiments is possible, a systematic analysis of the underlying physical effects would therefore require model modifications, and remains problematic to conduct within the studied ENM variants. A comparison with other coarse-graining approaches such as rotation-translation of blocks [34] (e.g., NOLB [35]), or with collective motions observed in molecular dynamics simulations [36], may provide additional valuable information to improve the model.

It can be furthermore interesting to discuss the effects of sequence- and distance-dependent stiffness in dynamical simulations of elastic networks, where conformational changes in a protein are directly resolved as over-damped relaxation motions of network residues (e.g., [37,38]). These descriptions include the full non-linear network dynamics beyond the harmonic approximation assumed in the analysis of normal modes, and allow dynamical probing of anisotropic responses of protein elastic networks generated by external perturbations or binding of ligands [39,40]. Effects of heterogeneous interaction parameters in those models have not yet been considered.

## Appendix A

### Appendix A.1. Tables of Stiffness Constants

For the sequence-specific model with a cutoff distance (sANM${}_{l_{c}}$) and for the model with sequence specificity and distance dependence (sdANM), we extracted the stiffness constants from tables provided in the paper by Dehouck and Mikhailov [15]. For sANM${}_{10}$, values from the file SuppTable3_sENM10.txt were used. For sANM${}_{13}$, values from the file SuppTable4_sENM13 were used. For sdANM, values from the file SuppTable5_sdENM.txt were used. We remark that Supplementary Tables S3–S5 (corresponding PDF files of stiffness tables) from the original publication do not contain information on the stiffness of bonded interactions (which was different for the models).

### Appendix A.2. Set of PDB Crystal Structures (Additional Information)

If, within the ATOM records of a PDB file, a specific amino-acid residue had alternative spatial coordinates of its atomic groups provided, only the first record was considered in building the corresponding elastic network model (i.e., from e.g., AALA, BALA, the latter entry was discarded).

### Appendix A.3. Analysis of NMR Protein Data and Comparison with Model Prediction

To evaluate the performance by the different model variants, we compared for a given protein the predicted pattern of residue fluctuations of its elastic network with the conformational variability derived from the ensemble of corresponding NMR models. The NMR data for a given protein was analyzed in the following way. Each model was superimposed (aligned) with the first NMR model (model 1) by minimizing the mean-square displacement (MSD). From this set of superimposed conformations, for each protein residue *i*, the average conformation ${\bar{R}}_{i}=\langle R_{i}\rangle$ was determined, and then the MSD from the average conformation was computed as MSD${}_{i}=\langle |R_{i}-{\bar{R}}_{i}{|}^{2}\rangle$, where 〈·〉 denotes the average over the aligned models. The comparison between model prediction and experiment was undertaken, at the residue level, by comparing MSF${}_{i}$ (see Section 2 in the main text) with MSD${}_{i}$, and, for a protein, by computing the correlation coefficient. The pattern of mean-square fluctuations MSF${}_{i}$ was always computed for the elastic network corresponding to the first model of a given protein. The underlying assumption is that ENM predictions are robust with respect to some changes in the network architecture (which would be the result of constructing the network for the different NMR models of the same protein) [21]. This assumption is justified by the imposed selection criteria of protein sets (see below).

### Appendix A.4. Set of PDB Solution NMR Protein Structures

Only data sets specified as proteins were considered. We omitted sets which had missing atoms/residues or unconventional residue types, less than 20 models, or less than 50 residues. Furthermore, if the ANM${}_{10}$ constructed for the first model of a protein data set had additional zero-modes in the spectrum, this data set was discarded (otherwise no additional zero-modes in all the considered models). It was also very important to get rid of intrinsically disordered proteins (IDPs) and proteins which have long intrinsically disordered regions. For those proteins, the elastic network description fails. Therefore, we have implemented a threshold for the conformational variability within the ensemble of models for a given protein. It was required that the overall root-mean-square displacement $\sqrt{\frac{1}{N}\sum_{i=1}^{N}{\mathrm{MSD}}_{i}}<2$ Å. After applying all the selection criteria, our data set contained 132 different NMR structures.

### Appendix A.5. Data Analysis

To classify the chemical specificity of a residue bead, the following categorization was applied. Hydrophobic: Alanine (Ala), Isoleucine (Ile), Leucine (Leu), Methionine (Met), Phenylalanine (Phe), Valine (Val), Proline (Pro), Glycine (Gly). Polar: Asparagine (Asn), Cysteine (Cys), Glutamine (Gln), Histidine (His), Serine (Ser), Threonine (Thr), Tryptophan (Trp), Tyrosine (Tyr). Charged: Arginine (Arg), Aspartic acid (Asp), Glutamic acid (Glu), Lysine (Lys).

To classify the location of a residue bead within secondary structural motifs, we have applied a simple scheme based on the information given in the PDB file of a protein. All residues listed in the HELIX record were categorized as part of an alpha-helix. All residues in the list of the SHEET record were grouped as part of a beta-sheet. All other residues were categorized as unstructured.
